# Unraveling the Hormonal and Molecular Mechanisms Shaping Fruit Morphology in Plants

**DOI:** 10.3390/plants14060974

**Published:** 2025-03-20

**Authors:** Muhammad Rafiq, Min Guo, Amna Shoaib, Jiaxin Yang, Siqing Fan, Haijing Xiao, Kai Chen, Zhaoqi Xie, Chunsong Cheng

**Affiliations:** 1 Jiangxi Key Laboratory for Sustainable Utilization of Chinese Materia Medica Resources, Lushan Botanical Garden, Chinese Academy of Sciences, Jiujiang 332900, China; rafiqsabqi@gmail.com (M.R.);; 2 Lushan Xinglin Institute for Medicinal Plants, Jiujiang Xinglin Key Laboratory for Traditional Chinese Medicines, Jiujiang 332900, China; 3 Department of Plant Pathology, Faculty of Agriculture, University of the Punjab, Lahore 54590, Pakistan

**Keywords:** fruit shape, phytohormones, fruit quality, cell division, molecular mechanism, genetic pathways

## Abstract

The importance of fruit shape studies extends beyond fundamental plant biology, as it holds significant implications for breeding. Understanding the genetic and hormonal regulation of fruit morphology can facilitate targeted breeding strategies to enhance yield, quality, and stress resistance, ultimately contributing to sustainable farming and nutrition security. The diversity in fruit shapes is the result of complex hormone regulation and molecular pathways that affect key traits, including carpel number, fruit length, and weight. Fruit shape is a quality attribute that directly influences consumer preference, marketability and the ease of post-harvest processing. This article focuses on investigations carried out on molecular, genetic and hormonal regulation mechanisms of fruit shape, color, maturation in fruit plants and key genetic pathways such as CLV-WUS and OVATE, as well as their roles in shaping non-climacteric fruits such as strawberries, grapes and raspberries. Plant hormones, especially abscisic acid (ABA) and indole-3-acetic acid (IAA), play a crucial role in enhancing desirable traits such as color and taste, while regulating anthocyanin synthesis and growth time. In addition, the dynamic interactions between auxin, gibberellin, and ethylene are crucial for the ripening process. Jasmonate enhances stress response, brassinosteroids promote ripening and cytokinins promote early fruit development. In addition, this review also studied the fruit morphology of species such as tomatoes and cucumbers, emphasizing the importance of the CLV-WUS pathway, which regulates the number of carpels through genes such as WUSCHEL (WUS), FRUITFULL1 (FUL1), and auxin response factor 14 (ARF14). The weight of fresh fruit is affected by microRNAs such as miRNA156, which emphasizes the importance of post transcriptional regulation. The involvement of transcription factors such as SISHN1, CaOvate, and CISUN25-26-27a further emphasizes the complexity of hormone regulation. Understanding these regulatory mechanisms can enhance our understanding of fruit development and have a profound impact on agricultural practices and crop improvement strategies aimed at meeting the growing global demand for high-quality agricultural products.

## 1. Introduction

The diversity of plant fruit shapes represents a significant evolutionary adaptation that enhances seed dispersal and survival in dynamic environments. Fruits are generally classified into two main categories: fleshy and dry. Fleshy fruits develop through both cell division and expansion, undergoing substantial changes in texture, color, and flavor during ripening, regulated by complex metabolic and hormonal processes [1,2]. In contrast, dry fruits do not exhibit such pronounced expansion or compositional transformations, and their developmental regulatory networks are comparatively well understood [2,3]. Fleshy fruits evolved from ancestral siliques through a conserved genetic network, developing diverse edible structures for seed dispersal. Examples include drupes (mango, cherry), berries (Schisandra, tomato, grape), pomes (apple, pear), hesperidiums (orange, lemon), and pepos (watermelon, pumpkin). In contrast, dry fruits lack a fleshy pericarp and are classified as dehiscent (legumes, capsules) or indehiscent (nuts, achenes, grains), relying on wind, water, or animals for dispersal [4,5].

Crop improvement and domestication have enhanced fruit diversity by selecting desirable traits, including shape and color. Squash varieties exhibit scallop and long crookneck forms, while tomatoes vary in shape, including round, ellipsoid, rectangular, heart, oxheart, and obovoid. Cucumbers are typically long cylindrical or round, and peaches are generally flat or round (Table 1). These fruit shapes serve as models for studying shape variation in Cucurbitaceae, Solanaceae, and Rosaceae fruit crops [6,7]. Fruit development in tomatoes unfolds through distinct stages: floral organ formation, cell division, cell expansion, and maturation. The initial stage lasts for 14 to 21 days, laying the foundation for the identity and shape of the flower. Subsequently, there is a two-week period of cell division leading to fertilization, followed by significant cell expansion, which can increase in size by more than 20 times. Maturity stabilizes size and shape, while triggering rapid biochemical changes such as changes in color and aroma [8]. The maturation process is subject to complex regulations and differs between climacteric fruits such as bananas and tomatoes, which mature through the production of ethylene after harvest, and non-climacteric fruits, such as grapes and strawberries, which ripen on the plant without undergoing significant post-harvest changes [9,10]. The balance of sugar and acid has a crucial impact on flavor, and indicators such as titratable acidity play a key role in determining maturity [11]. This review explores the genetic, hormonal, and environmental interactions that regulate fruit shape diversity, providing insights into the molecular mechanisms underlying fruit development. It examines the genetic control and hormonal regulation of fruit shape, color, taste, and ripening processes.

**Table 1:** Variation in fruit shapes among different fruit crops.

Fruit Shape	Examples	Fruit Family	Reference
Spherical	*Malus domestica*, *Citrus sinensis*, *Solanum lycopersicum*	Rosaceae, Rutaceae, Solanaceae	[12]
Oval	*Musa paradisiaca* Linn., *Vitis vinifera* L., *Carica papaya*	Musaceae, Vitaceae, Caricaceae	[13]
Elongated	*Cucumis sativus Solanum melongena Capsicum*	Cucurbitaceae, Solanaceae	[14]
Irregular	*Fragaria x ananassa*, *Ficus carica*, *Actinidia deliciosa*	Rosaceae, Moraceae, Actinidiaceae	[15]
Oblong	*Mangifera indica*, *Persea americana*, *Prunus domestica*	Anacardiaceae, Lauraceae, Rosaceae	[16]
Globular	*Vaccinium koreanum*, *Vitis vinifera* L., *Prunus avium*	Ericaceae, Vitaceae, Rosaceae	[17]
Pear-shaped	*Pyrus communis*, *Carica papaya Guava*	Rosaceae, Caricaceae, Myrtaceae	[18]
Spherical	*Malus pumila*, *Citrus sinensis*, *Solanum lycopersicum*	Rosaceae, Rutaceae, Solanaceae	[12]

## 2. Molecular and Genetic Regulation Mechanisms of Fruit Shape

In recent years, with the introduction of innovative tools such as the tomato analyzer developed by [19], there has been an increasing trend in determining the diversity of fruit shapes. It categorizes tomato fruit shapes into eight different types, such as long, bovine heart, heart-shaped, flat, rectangular, circular, inverted spherical, and elliptical [20]. In contrast, gourd family crops such as cucumber, melon, and watermelon typically exhibit more uniform shapes, mainly cylindrical or elliptical [21]. The morphological occurrence and development of fruit shape are influenced by various internal factors, including protein interactions, regulatory genes, and external environmental conditions. This study classified various fruit shapes and delved into the regulatory mechanisms that drive their formation.

### 2.1. Carpel Number

**Figure 1:**
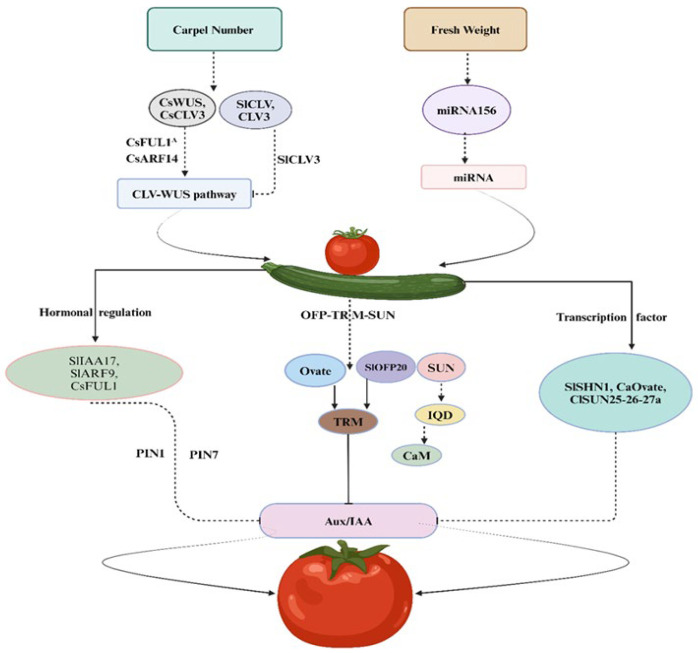
*CLV-WUS* and *OVATE*-based model pathway and role of associated genes in fruit traits.

The carpel number (CN) stands as a pivotal fruit trait among vegetables that effects both shape and size of the fruits. In the natural course, typically fruits produce two or more than two carpels, with variations attributed to domestication and mutations. In Arabidopsis, research shows that shared regulatory factors are important to determine *CN*, *WUSCHEL (WUS)* and *CLAVATA (CLV)* involved in negative feedback mechanisms that regulate the floral organs’ number and meristem size (Figure 1). Genetic mutation belonging to the CLV family leads to an increased number of undifferentiated cells mostly present in the central area, leading to expansion of the meristem cells and eventually resulting in shaping the fruit [22]. Larger floral meristems correspond to increased CN and fruit width [23]. Significantly, in higher plant *CLV-WUS* pathways, function is conserved [24], regulating the fruit CN in plants belonging to Brassicaceae Cucurbitaceae and Solanaceae families [25,26,27]. In cucumbers, transcription factors (TFs), *CsARF14*, and *CsFUL1A* genes (*FRUITFULL*-like *MADS*-box) regulate the CsWUS-*CsCLV3* pathway, playing an important role in the cucumber CN-regulation mechanism [25]. It is observed in tomato plants that a loss of function mutation in *SlCLV3* genes leads to an increase in the size of the fruit due to an increased number of locules [26,28]. Moreover, the upregulation of gene (*WOX1 TF*), belonging to the *SlWUS* family, affects fruit shape and carpel number [29]. The locule number (LC) in tomatoes is regulated by the *WUS* gene, while the *FAS* gene encodes a regulator that influences CLV3 expression, collectively controlling fruit locule development, mutations in *LC* or *FAS,* resulting in a high yield of locule count [30]. Besides, the transcriptional regulation of non-coding RNAs (long or small) shows a critical role in fruit shape development. Locule number in tomato can be altered by overexpression of *MIR156* (156 MicroRNA) [31]. In rapeseed, *CLV1* disruption may result in a trilocular phenotype [27] (Table 2). Furthermore, a plant-specific TF belonging to the YABBY family includes a number of members capable of increasing the locule number and influencing the flat fruit shape in tomatoes [20,32].

**Table 2:** Genes associated with fruit shape development.

Gene	Function	Fruit Species	Reference
*FASCIATED (FAS)*	Cell division regulation	Tomato, Capsicum	[33]
*OVATE (O)*	Fruit shape determination	Tomato	[34]
*SUN*	Cell expansion control	Tomato, Pepper	[35]
*SGR*	Cell wall metabolism	Tomato	[36]
*LCY-B*	Carotenoid biosynthesis	Tomato, Pepper	[37]
*CsFUL1A*, *CsARF14*	Regulates carpel number	Cucumber	[25]
*SlCLV3*	Increases locule number and fruit size	Tomato	[26,28]
*SlWUS (WOX1 TF)*	Affects fruit shape and carpel number	Tomato	[29]
*CLV1*	Disruption induces a trilocular phenotype	Rapeseed	[27]
*YABBY TFs*	Enhances locule number and promotes flat fruit shape	Tomato	[20,32]

### 2.2. Fruit Length

The phenotypic trait of fruit length (FL) is significantly influenced by diverse factors, treated as a quantitative trait, and has been the subject of investigation through quantitative trait loci (QTL) mapping, leading to the identification of various relevant loci. A multitude of candidate genes linked to FL, including *fl3.2*, *mfl3*, *fl7.1*, *fl4.1*, *FS3.2*, *FS3.3*, and *fs10.1*, have been discerned [38,39,40]. Moreover, an abundance of genes and regulatory pathways affecting FL have been unveiled. In cucumbers, genes like short fruit (sf1) and *CsFUL1* have been reported for FL regulators [41,42]. The sf3 mutation in cucumbers is associated with the candidate gene *CsKTN1*, a homolog of *KTN1*, which was first identified in *A. thaliana* and is involved in the regulation of nuclear transport; this gene also encodes a katanin p60 subunit and plays a role in influencing FL regulation [43]. In tomatoes, regulatory factors such as Tonneau1 recruitment motif (TRM), *SUN* and *Ovate* family proteins (*OFP*) have individual or collective effects on FL. The identification of the regulatory gene *Ovate* in pear-shaped tomato fruit by Liu et al. marked a significant discovery [44]. Extensive research on plant-specific *TFs OVATE* and *SlOFP20* has shown their impact on tomato fruit size and shape. *OVATE* induces pear-shaped fruit, while *SlOFP20* regulates floral organ and pollen tube development by modulating brassinosteroid (BL) and gibberellin (GA) signaling pathways [45].

**Figure 2:**
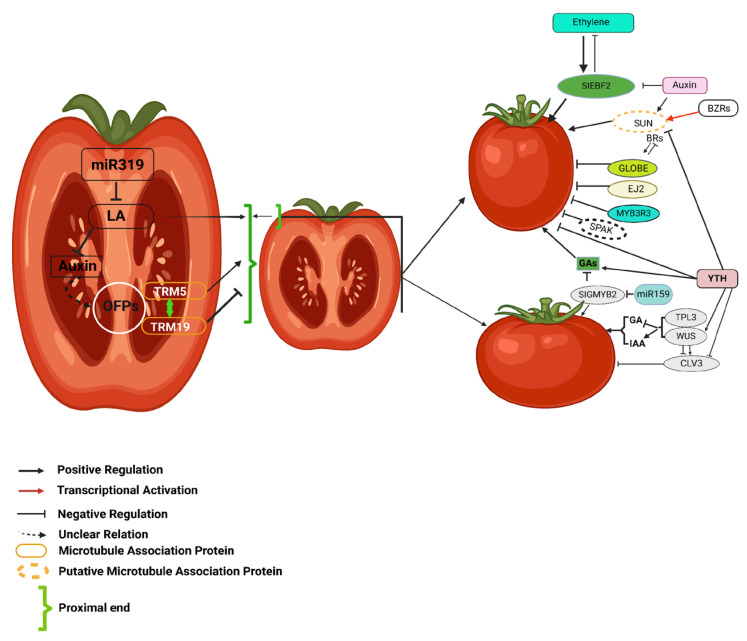
This figure illustrates the regulatory network of hormonal and genetic interactions influencing tomato fruit morphology. Auxins, gibberellins (GAs), ethylene, and brassinosteroids (BRs) coordinate fruit shape and size by modulating key genetic regulators. CLV3, controlled by WUS, TPL3, and hormonal signals, determines locule number. The interplay between SUN, GLOBE, EJ2, and microtubule-associated proteins, regulated by BRs and ethylene, further refines fruit morphology. Additionally, miR159 and miR319 modulate transcription factors affecting auxin and GA pathways. This intricate network highlights the coordinated influence of hormonal and genetic factors in shaping tomato fruit development. The figure is adapted with slight modifications from Li et al. (2023) [49].

Research involving the overexpression of *AtOFP1* in Arabidopsis, like the overexpression of *OVATE* and *SlOFP20* in tomatoes, suggests a shortened length of floral organs [44]. Tomato fruit morphology is controlled by a complex regulatory network of hormonal signaling and genetic interactions. The fruit shape and size are modulated by auxins, gibberellins (GAs), ethylene, and brassinosteroids (BRs) through key regulatory pathways (Figure 2). In tomatoes, egg-shaped mutations induce changes in fruit cell-division patterns, affecting the number of cells in both proximal and distal directions [33]. In addition, the locus inhibitors of *OVATE 1* (*SOV1*) enhance the impact of *OVATE* mutations [46]. Furthermore, due to the overexpression of homologous *OFP* genes (*AtOFP15*, *AtOFP16*, *AtOFP13*, and *AtOFP18*) in Arabidopsis, the reduction in siliqua length was exaggerated [47]. These genes are also responsible for regulating fruit shape, indicating that this gene family may also affect organ shape in other plant species [48].

Although the molecular mechanism of *OVATE* is not clear yet, it has been shown that it works together with some microtubule-associated proteins, such as *TRM* [50]. *SlOFP20* and *OVATE* engage with the *TRM* M8 motif by the *OFP* domain. When co-expressed *OVATE* or *SlOFP20* with *SlTRM*3/4 induce the relocation of *TRMs* and *OFPs* from microtubules toward cytoplasm, this indicates that the *OFP*–*TRM* protein complex is important for organ growth and cell division by maintaining a dynamic equilibrium among micro tubular and cytoplasmic localization [34,51]. The *CaOvate* gene suppression in peppers resulted in an elongated fruit shape, indicating the role of *OFP* families in regulating fruit size [52]. *SUN* is a member of the family (IQ 67 domain (*IQD*)) that encodes a protein involved in Calmodulin (CaM) binding [46,53]. *SUN* in melons, cucumbers and tomatoes is known as a fruit length (FL) and shape regulator [33,53,54,55]. The *IQD*, a conserved region of 67 amino acids consisting of three regularly spaced IQ motifs that facilitate binding of CaM in the occurrence of Ca^2+^ [56,57], triggers changes in fruit shape through *SUN* during the cell division stage, 7–10 days post-pollination [58]. This change involves increased cell division and elongation beside the proximo-distal axis, allowing the regulation of fruit structure by affecting microtubule dynamics [59]. Lazzaro et al. (2018) proposed a model illustrating the interaction between *SUN*, *OFP*, and *TRM* in tomato fruit-shape regulation, particularly in connection with microtubule development [52]. Additionally, plant-specific *Rho GTPases* (*ROPs*) play an important role in organizing microtubules and influencing the cytoskeleton to determining the absolute shape of a cell [60].

Several *IQD* proteins have been identified as key facilitators of *ROP* domains’ formation, contributing to the regulation of cytoskeletal structure at the plasma membrane. Within this interaction network, the upregulation of *TRMs* and *SUNs*, or the downregulation of *OFPs*, can lead to an elongation of tomato fruit [52]. Research shows that *SUN*/*IQD TRMs* and *OFPs* collectively influence the microtubule activity, consequently impacting the shape of the fruit. *SUN* family proteins (*Csa1G575000*, *CmSUN-14*, *CsGy1G026840.1*, and *Cla011257*) are known as FL regulators in cucurbitaceous crops [21,52,54,61]. Further investigation shows that the *Cla011257* gene impacts the ovary before anthesis to influence FL during the development period [62]. *MADS*-box transcription factors (*TFs*) are closely linked with development of a plant, significantly contributing to fruit formation [63]. *FUL* is a vital TF member of the *MADS*-box family that plays an important role in regulating FL in various plants. For instance, *MBP7*/*FUL2* in tomatoes regulate the shape of fruits by modulating cell division and enlargement. Fruit fails to elongate after fertilization as a result of knocking out *FUL* genes and encoding *MADS*-box proteins in different species, with a significant reduction in valve size [52,64]. In cucumbers, *CsFUL1A* acts as a negative regulator by inhibiting auxin transport and cell division, thereby affecting FL. *CsFUL1A*, another *MADS*-box *TF*, binds to the *CArG*-box, regulating cell division and expansion by repressing the expression of auxin transporters *PIN1* and *PIN7*, which reduces auxin accumulation and controls FL [42].

### 2.3. Fruit Weight

The fruit weight (FW) is intricately linked to the size of the fruit. During the breeding of vegetables, multiple loci governing FW have been identified (Table 1). FW, being a quantitative characteristic, is under the control of numerous loci [65]. In Solanaceae vegetables and especially in tomatoes, several genes, including *fw1.1*, *fw11.2*, *fw3.3*, *FW3.2*, and the Cell Size Regulator (*CSR*), involved in regulating FW have been successfully cloned [66]. Notably, *FAS* and *LC* homologous to *YABBY2* and *WUS,* respectively, have been identified as genes influencing the locule number (CN) to enhance FW [23,30]. In the Cucurbitaceae family, a number of loci are associated with FW. Cucumbers, for instance, have three QTLs described such as *fw6.1*, *fw2.1* and *fw4.1* [67]. In melons, FW has been regulated by genomic regions *FWQM11* and *FWQM8* [68]. A *CYP78A* gene with the subfamily *KLUH* primarily controls the size of organs in Arabidopsis. Various members of subfamily *CYP78A* are from vegetables, such as *GmCYP78A72* (soybean), *BnaA9.CYP78A9* (rapeseed), *CaKLUH* (pepper), and *SlKLUH* (tomato). *GmCYP78A10* have been said to regulate FW, and a recent research also indicated that the upregulation of the transcription factor gene *SHINE1* (*SlSHN1*) can lead to a decline in the FW of a tomato [69,70,71].

### 2.4. Regulation of Fruit Shapes by Hormone

Phytohormones, responsive to both external and endogenous stimuli during a plant’s development, play a crucial role in various fruit development stages, influencing growth and eventually determining the fruit shape and size [72,73]. Among the numerous plant hormones, comprising cytokinin (CK), ethylene, gibberellin (GA), abscisic Acid (ABA) and auxin, several have been identified for their impact on fruit shape (Table 3). Auxin, in particular, plays a pivotal function in the fleshy fruit development [7,74]. In the study of different cucumber inbred lines by Liu et al. (2020), endogenous hormone content, especially indole-3-acetic acid (IAA), was positively correlated with fruit size and cell growth at different developmental stages [7]. Processing the *LA1589* near the isogenic line of eggplant, which is characterized by slender pear-shaped fruits and ovaries, early flowering with auxin (2,4-D) can lead to an increase in cell size and quantity near the fruit or ovary [75]. The gene *CsYUC10b*, which synthesizes auxin, is associated with fruit arch, and its upregulation induces the development of straight fruit [76]. In addition, *SlIAA17* is a transcriptional repressor of auxin/indole-3-acetic acid (Aux/IAA) and is associated with an increase in tomato fruit size [77].

Members of the auxin response factors (*ARFs*) family, such as *SlARF9* in tomatoes and *BnaA9.ARF* in *Brassica rapa*, have been identified as regulators of fruit size [69]. Apart from auxin, additional hormones collaborate with specific regulatory factors to influence fruit shape [78]. Cytokinins (CKs), primarily responsible for regulating cell division in plants, exhibit a positive relationship with fruit cell division activity [54,79,80]. The gene responsible for CK biosynthetic (*CYP735A*) has the capacity to alter cell size and biomass accumulation, thereby impacting size of the fruit [81]. In cucumbers, initial stages of fruit development are dependent on trans-Zeatin riboside (tZR), particularly in the early ovary development phase, influencing cell division. Zeatin (ZT) content, on the other hand, increases in the early stages after flowering, facilitating the horizontal expansion of cells [7]. Gibberellin (GA) plays an important role to stimulate fruit and seed development and regulate flowering [54]. The GA application induces cell increase and can result in parthenocarpy [82]. In cucumbers, GA promotes cell expansion during fruit development [83], and 9–12 days after anthesis, at the mid-to-early stage, counteracts indole-3-acetic acid (IAA), potentially impeding fruit enlargement by delaying cell division. The accumulation of GA during fruit development in tomatoes aligns with the direction of cell division in the early stages [84], while ethylene is predominantly associated with fruit ripening in tomatoes [85], and recent reports suggest its regulatory role in cucumber fruit length.

The ethylene-based content also plays an important role in influencing plant development. The enzyme 1-aminocyclopropane-1-carboxylate synthase 2 (ACS2) is important for catalyzing ethylene biosynthesis [84], and the cucumber mutant with decreased ethylene, acs2, exhibits a reduction in fruit length. Conversely, in *sf1* mutants, an excess of ethylene can result in the same phenotype [41]. Hence, an imbalanced ethylene concentration, whether excessive or insufficient, can impact fruit length. The homologous ACS gene, *CmACS7*, has been linked to the round shape of melon fruits [86]. These findings indicate that the function of dosage-dependent ethylene in Cucurbitaceae is comparatively conserved. In a distinct research based on tomatoes, a number of Aux/IAA-like genes such as *DR1*, *DR3*, *DR4*, and *DR8*, associated with indole-3-acetic acid (IAA), were controlled by ethylene [87]. Abscisic acid (ABA), known as plant growth inhibitory hormone [88], is noted to have an impact on fruit size and cell shape in tomatoes, as evidenced by the small sized fruit and altered shape of cells in the ABA-deficient mutant [88,89]. The research highlights the synergistic effects or potential antagonistic effect between plant hormones, collectively governing development and ultimately effecting the shape of fruits.

**Table 3:** Hormones involved in fruit development.

Hormone	Function	Effects on Fruit Shape	Reference
Auxins	Cell elongation, fruit growth	Promotes elongation, determines fruit shape	[90]
Cytokinins	Cell division, cell differentiation	Regulates fruit size, influences shape development	[91]
Gibberellins	Cell elongation, seed germination	Stimulates elongation, affects fruit size and shape	[92]
Abscisic Acid	Seed dormancy, stress response	Regulates fruit maturation, influences shape and ripening	[93]
Ethylene	Fruit ripening, senescence	Controls fruit ripening, affects texture and shape	[94]

## 3. Role of Hormones in Fruit Ripening

Traditionally, comparative genomic analyses in hot peppers (*Capsicum* spp.) and tomatoes as models for fruits, respectively, reveal commonalities in gene expression. Specifically, genes encoding transcription factors like tomato *AGAMOUS*-like 1 (*TAGLI*), non-ripening (NOR), ripening inhibitor (RIN), and components related to the ethylene signaling pathway are identified as shared features in both fruit types [95]. The presence of *MADS*-box genes in both categories further suggests overlapping molecular regulatory processes in the maturing of climacteric and also in non-climacteric fruits [96]. Plant hormones play a pivotal role in regulating fruit ripening [97,98]. The combined function of hormones such as cytokinin, gibberellins and auxin helps in normal growth for fruit and the shape of the fruit, even if they have no fertilization; this process is known as parthenocarpy. The subsequent discussion provides an overview of plant hormones involved in the ripening of non-climacteric fruits, specifically in grape, strawberry and raspberry, along with a discussion on potential hormone crosstalk [99]. The subsequent section also discusses various hormones and their associated genes, highlighting their roles in fruit development, as summarized in Table 4.

**Table 4:** Key plant hormones, their roles, and associated genes in fruit development.

Hormones	Role in Fruit Development	Associated Genes	Reference
Auxins (IAA)	Regulates fruit initiation, cell expansion, and ripening	*ARF14*, *ARF7*, *ARF8*, *TIR1*, *FUL1*	[77]
Gibberellins (GA)	Promotes fruit growth, elongation, and seed development	*GA20ox*, *GA3ox*, *RGA*, *GID1*	[100]
Cytokinins (CKs)	Stimulates early fruit development and delays senescence	*IPT*, *CKX*, *ARR*-*B*, *LOG*	[101]
Abscisic Acid (ABA)	Controls ripening, color development, and anthocyanin synthesis	*NCED*, *PYR/PYL*, *SnRK2*, *ABF*	[102]
Ethylene	Induces ripening, softening, and aroma production	*ACS*, *ACO*, *EIN2*, *ETR1*	[103]
Jasmonates (JA)	Enhances stress responses and influences ripening	*JAZ*, *MYC2*, *COI1*	[104]
Brassinosteroids (BRs)	Modulates fruit size, weight, and ripening	*BRI1*, *BZR1*, *DWF4*	[105]
Salicylic Acid (SA)	Regulates defense mechanisms and fruit quality traits	*NPR1*, *TGA*, *PR1*	[106]

### 3.1. Abscisic Acid (ABA)

One of the most important phytohormones is abscisic acid (ABA), which significantly influences various plant processes, particularly stress responses, seed dormancy and fruit development [107]. In strawberries and grapes, ABA acts as an important part in the ripening process, especially in the absence of ethylene spikes characteristic of climacteric fruits [93]. Historically recognized as being responsible for grape berry ripening, ABA impacts sugar accumulation, coloration, and softening, essential traits for desirable fruit quality [108]. ABA level during early stages of fruit development is typically low but increases significantly as ripening progresses. This interplay highlights how enhanced flavonoid levels contribute to the overall quality and appeal of the fruit, illustrating ABA’s multifaceted role in fruit ripening and quality enhancement.

### 3.2. Regulation of Fruit Quality by Exogenous ABA

The influence of ABA goes beyond simple maturation; it also increases the anthocyanin content in strawberries and grapes by upregulating enzymes such as anthocyanin synthase (*ANS*) and glucosyltransferase (*GT*) [109]. This relationship emphasizes the regulatory role of ABA not only in the ripening process, but also in fruit color development and nutritional value. In strawberries, ABA treatment accelerates softening, enhances coloration, and increases ethylene production while activating phenylalanine ammonia-lyase (PAL), a key enzyme in the phenylpropanoid pathway that synthesizes flavonoids and other metabolites [109]. Additionally, ABA treatment elevates l-ascorbic acid levels, highlighting its essential role in improving fruit quality. Interestingly, sucrose-induced maturation is associated with an increase in ABA biosynthesis, indicating a synergistic relationship between sugar levels and ABA activity [110]. In “flame seedless” grapes, the application of ABA can increase anthocyanin content, improve color, and accelerate softening—a key factor in consumer acceptance and marketability [111]. These findings emphasize the regulatory role of ABA in regulating the essential metabolites that define mature fruits, creating a feedback loop where these metabolites can affect ABA biosynthesis itself, further illustrating the complexity of fruit ripening mechanisms.

### 3.3. The Effect of Inhibitors on ABA Biosynthesis and Fruit Characteristics

Exploring the dynamics of ABA during fruit ripening and revealing the effect of inhibitors on its synthesis, a type of ABA synthesis inhibitor treated with fluoroketone leads to a decrease in ABA levels and helps maintain fruit texture under storage conditions [112]. In strawberries, the use of dehydroguaiacolic acid (NDGA), an NCED enzyme inhibitor, not only reduces ABA content but also prevents red coloration in fruit containers, indicating that the enzyme plays a critical role in fruit ripening [113]. This observation reinforces the importance of ABA in forming ideal fruit characteristics. By studying the expression of the key gene *FaNCED1* in ABA synthesis, the relationship between ABA and its biosynthesis was further elucidated. In fruit tissue sucrose culture experiments, *FaNCED1* expression and subsequent ABA accumulation indicate that this gene is controlled by the accumulation of metabolites such as sucrose during strawberry ripening [114]. This complex interaction illustrates how metabolic signals regulate hormone levels and establish a complex regulatory network during fruit development.

#### 3.3.1. Elucidation of ABA Signaling Pathway

Recent advances in understanding the ABA signaling pathway, particularly in Arabidopsis, have identified two key pathways: the “ABA-ABAR-WRKY40-ABI5” ABA and PYR/PYL/RCAR-PP2C-SnRK2 pathways [115,116,117]. In the first pathway, the binding of ABA to the PYR1 receptor activates sucrose non-fermentation-associated kinase 2 (SnRK2) through inactivation of phosphatase 2C (*PP2C*) protein. This signaling cascade subsequently activates ABA response element-binding transcription factors (AREB/ABF), leading to the expression of various downstream genes, including genes related to NADPH oxidase and ion channels [88,116]. In contrast, another pathway begins at the ABAR receptor, which interacts with the WRKY40 transcription factor. This interaction occurs under elevated ABA conditions and promotes upregulation of ABA responsive gene expression, which is associated with key transcription factors such as ABI4 and ABI5 [118,119,120]. Characterizing these pathways in crops such as grapes and strawberries can provide a deeper understanding of how ABA signaling affects fruit ripening.

#### 3.3.2. The Agronomic Benefits of ABA on Fruit Quality

In grape varieties such as ’Kyoho’, the expression of a PYR1-like gene (*VlPYL1*) increases during fruit development, which is associated with improved ABA sensitivity and fruit quality, particularly in terms of anthocyanin content [121]. The overexpression of this gene enhances the transcription of ABA responsive genes, creating an environment conducive to high-quality fruit development. Similarly, in strawberries, downregulation of FaABAR hinders ripening, highlighting the importance of this receptor in fruit ripening [122]. Ultimately, understanding the regulatory role of ABA in fruit ripening is of great significance for agriculture. By utilizing the influence of ABA on basic fruit quality attributes such as color, hardness, and nutritional composition, growers can optimize ripening conditions to improve harvest quality. Innovative strategies have been proposed to improve fruit quality, extend shelf life and enhance consumer satisfaction in response to the ABA signaling pathway [48]. A deeper understanding of the complex relationship between ABA and fruit development can pave the way for advances in horticultural science and sustainable agriculture, ensuring that the growing demand for high-quality agricultural products is met while improving the economic feasibility of farmers [123].

In short, the regulatory function of ABA goes beyond academic interest. It has transformative potential for agricultural practices, promoting healthier diets and sustainable food systems. The in-depth understanding of the complex interactions between ABA and metabolic pathways emphasizes the necessity of continuous research. Understanding the role of ABA in fruit ripening can lead to innovative agricultural technologies that improve fruit quality and sustainability. With the increasing demand for high-quality agricultural products, strategic ABA applications may provide solutions that benefit consumers and producers, thereby fostering a resilient agricultural sector. Utilizing these insights can ensure a sustained supply of nutritious and attractive fruits for a growing population.

### 3.4. Indole-3-Acetic Acid (IAA)

Auxins, especially indole-3-acetic acid (IAA), are key plant hormones that can coordinate various developmental processes, including fruit development [124]. Its role in climacteric and non-climacteric fruits has been extensively studied, highlighting its dual function as a growth promoter and regulator of development time. The involvement of auxin in fruit development is complex, involving interactions with other hormones, transport mechanisms, and gene expression regulation [125].

#### 3.4.1. The Role of Auxin in Fruit Growth Kinetics

Auxin is mainly synthesized through a two-step process, involving tryptophan as a precursor. In Arabidopsis, this biosynthetic pathway is promoted by the *YUCCA* (*YUC*) family, containing flavin monooxygenases, and the Arabidopsis tryptophan aminotransferase 1 (*TAA1*) family. Indole-3-pyruvic acid is formed through tryptophan conversion with the help of the TAA1 protein family, followed by conversion to YUC protein and IAA. Studies on grapes have shown that this biosynthetic pathway is active throughout the entire berry development process, with specific *TAR* and YUCCA genes showing high levels of expression during early fruit development and the onset of ripening [126]. This indicates that auxin synthesis is crucial during critical developmental stages, demonstrating its role in establishing the fruit setting and subsequent growth. In non-climacteric fruits such as strawberries, auxin plays a crucial role in the development of achenes and flower beds. The main source of auxin in strawberries is achene, which promotes the growth of flower beds. Removing achenes from immature containers can hinder growth and expansion, leading to upregulation of maturation-related genes [127,128]. This indicates that auxin is not only crucial for fruit size, but also for ripening time and overall fruit quality.

As is well known, auxin can stimulate fruit growth, so its impact on ripening is more subtle. In grapes, the high concentration of IAA in young fruits decreases with the maturation process, indicating that auxin may inhibit sugar accumulation and anthocyanin production, leading to a complex relationship of delayed maturation [129]. Throughout the entire maturation process, the regulation of IAA levels highlights the dual role of this hormone as a growth promoter and maturation kinetics regulator.

In strawberries, transcriptional analysis of fruit treated with auxin showed downregulation of genes involved in flavonoid biosynthesis, aroma production, and cell wall modification. For example, genes encoding chalcone synthase, alcohol acyltransferase and pectin lyase were significantly affected by auxin treatment, indicating that it not only affects growth but also directly affects metabolic pathways associated with maturation [10].

#### 3.4.2. Auxin Transport and Internal Balance

The polar transport of auxin is promoted by specific transport proteins, including *AUX*/*LAX* and *PIN* proteins, which play a crucial role in establishing the auxin gradient required for directed growth and development. In grapevines, radiolabeled IAA studies revealed a basal distribution pattern in the skin cells, attributed to the activity of the speculated *VvPIN* protein. It is worth noting that during the process of young fruit abscission, the expression of several *VvPIN* genes decreases, indicating that auxin homeostasis is crucial for fruit setting [130].

In strawberries, auxin transporters exhibit developmentally specific transcription patterns, indicating their role in fruit growth and maturation. The identification of 10 FvPIN genes and *4 FvAUX/LAX* genes in the genome of forest strawberries emphasizes the genetic basis of auxin transport in this species [131]. In addition, the expression of these genes at different stages of fruit development indicates that auxin transport dynamics are essential for the development of both achenes and flower beds.

#### 3.4.3. Auxin Binding and Metabolism

The homeostasis of auxin is also regulated through binding processes, which activate or deactivate IAA. The IAA amide synthase (GH3) family plays a crucial role in this process, promoting the binding of IAA to amino acids, thereby affecting its availability and activity in plants. For example, in peaches, a potential IAA amide hydrolase was observed to remain upregulated during fruit ripening, similar to IAA-Leucine RESISTANT 1 (*ILR1*) in Arabidopsis, indicating the existence of a mechanism regulating auxin levels at this critical stage [132]. Under the stimulation of exogenous auxin, the expression of VvGH3.1 in grapes is associated with berry ripening, highlighting the importance of auxin binding in regulating fruit ripening [97]. The balance between IAA binding and hydrolysis seems crucial for maintaining auxin levels that are beneficial for fruit ripening and overall quality.

The interaction between auxin and various other plant hormones, particularly abscisic acid (ABA), ethylene, and gibberellin (GA), is crucial for fruit development and maturation. For example, in climacteric fruits such as tomatoes, auxin interacts with ethylene to regulate the ripening process [133]. The *RIN* (*RIPENING INHIBITOR*) gene is a *MADS*-box transcription factor that responds to auxin and plays a central role in this interaction. Recent studies have shown that RIN is optional in the early stages of fruit ripening and suggest that auxin signaling may be compensated by other *MADS*-box proteins during this stage [134].

In strawberries, the interaction between auxin, ethylene, and ABA also affects ripening kinetics. As is well known, ethylene increases the expression of auxin responsive genes, making the regulatory network that controls fruit ripening more complex. The balance between these hormones may determine the timing and quality of strawberry ripening and have an impact on commercial fruit production.

#### 3.4.4. Genetic Regulation of Auxin Signaling Pathway

Transcription factors involved in auxin signaling, such as auxin response factor (*ARF*) and *F-box* protein, are key mediators of a plant’s response to auxin. In cucumbers, two *F-box* auxin receptors, *CsTIR1* and *CsAFB2*, were identified, and their expression was highest in young fruit tissues [27]. In transgenic tomato lines overexpressing these genes, monosexual fruit development was observed, emphasizing the potential for manipulating auxin signaling to promote fruit setting and development. In strawberries, the differential expression of ARF throughout the entire maturation stage indicates a complex regulatory framework, in which not all ARF genes have a consistent response to auxin levels. For example, the comparative expression patterns of *ARF1* and *ARF4* during ripening indicate that specific *ARF* genes may promote or inhibit fruit development, depending on the developmental environment [135].

Although substantial progress has been made in understanding the role of auxin in fruit development, there are still many issues that need to be addressed. Further clarification is needed on the genetic and molecular mechanisms of auxin biosynthesis, transport, and signaling in non-climacteric fruits. Specifically, exploring the interactions between auxin and other hormones during fruit development and ripening remains a promising field for future research. The transcription factors that mediate auxin response, such as auxin response factor (*ARF*) and *MADS*-box proteins, particularly their interactions with other hormone pathways, deserve further investigation. The use of advanced molecular technologies such as CRISPR-based gene editing and RNA interference can provide new insights into the regulatory networks that affect fruit development, ultimately improving fruit quality and supporting sustainable agricultural practices.

### 3.5. Gibberellins

Gibberellin (GA) is a cyclic diterpenoid compound that is important in many growth processes, such as cell division, seed germination, flower induction, fruit growth, and elongation [126]. There are multiple GAs present in plants, with only a few having biological activity [136,137]. The balance of bioactive gibberellins is maintained through synthesis and inactivation processes, mainly mediated by enzymes such as gibberellin 2-oxidase (*GA2ox*) and gibberellin 3-oxidase (GA3ox) [138]. Thompson (1969) documented preliminary observations on the effects of exogenous GA application on strawberry receptacle development, and subsequent studies linked GA to fruit ripening, particularly in strawberries [139]. During the development of strawberry fruit, it has been found that the levels of bioactive GA increase, especially in stages 1, 3, and 4, with GA4 concentration reaching its peak during the white development stage.

#### 3.5.1. The Mechanism of Action of Gibberellin in Fruit Growth

The mechanism by which GAs affect fruit growth involves complex genetic and molecular pathways. Key genes related to the GA pathway, such as *DELLA*, *FaGID1c*, and *FaGID1* (*GIBBERELLIN-INSENSITIVE DWARF1b*), as well as proteins such as FaRGA (GA REPRESSOR), are upregulated in strawberry receptacle tissue during development [136]. It is worth noting that *FaGID1c* exhibits GA binding, interacts with *FaRGA* in vitro, and enhances GA response when expressed ectopically in Arabidopsis. When gibberellin stimulates overexpression of the transcription 2 (*FaGAST2*) gene in transgenic strawberries, it reduces fruit size, indicating that *FaGAST2* is associated with cell elongation and fruit size regulation [140]. In contrast, silencing *FaGAST2* resulted in the increased expression of *FaGAST1* without altering cell size, indicating complex interactions between these transcription factors in determining fruit cell size and development.

#### 3.5.2. Gibberellins in Viticulture

The application of exogenous GAs has garnered significant attention in viticulture, particularly regarding grape development. Pre-bloom GA3 application has been shown to foster seedlessness and increase the size of berries in seedless grapevines [141]. Transcriptome sequencing studies have indicated a potential role for grapevine miRNAs in berry development and responses to environmental conditions [142]. Furthermore, GA application in the ’Kyoho’ grape stimulated flower opening, facilitated fruit coloring, and led to seed abortion [143]. In Rubus genus, the role of GAs in fruit development is not well studied; some studies indicate that GA application can induce asexual fruit growth in cloudberry (*Rubus chamaemorus* L.) [144] and affect flower numbers in raspberries [145]. Future research should focus on elucidating the complex regulatory networks governing GA action in fruit development, including advanced molecular techniques like CRISPR and RNA interference, to enhance our understanding of GAs’ influence on fruit growth and ripening.

### 3.6. Ethylene

In recent years, the involvement of ethylene fruit ripening (especially non-climacteric fruits) has attracted widespread attention. The results of various studies indicate that it plays an important role in the ripening process of different grape varieties, with the peak of ethylene occurring before grape ripening [146]. The use of ethylene receptor inhibitor 1-methylcyclopropene (1-MCP) leads to a reduction in ripening-related factors and berry size in Cabernet Sauvignon grapes, such as anthocyanin accumulation [147]. On the other hand, ethylene treatment increased berry size and was associated with high expression levels of xyloglucan endoglucanase (XTHs), polygalacturonase, aquaporin, elastin, and cellulase [148]. On the contrary, the application of 1-methylcyclopropene prior to its presence reduced ABA levels in Muscat Hamburg grapes, indicating a possible interaction between ABA and ethylene during maturation [149]. In *Fragaria x ananassa* (strawberry), the concentration of ethylene is relatively low, and its yield varies depending on the stage of fruit development. The ethylene content is moderate in green fruits, lower in white fruits, and significantly increases during the red stage of fruit ripening. The increase in ethylene production during the red fruit stage is consistent with the increase in respiratory rate, like the maturation pattern observed in climacteric fruits [10].

#### 3.6.1. Production Modes of Ethylene in Different Fruits

Raspberry fruit exhibits a unique ethylene production mode. Unlike fruits such as strawberries and grapes, strawberries and grapes reach their peak ethylene activity in the early stages of fruit development [150], while raspberry’s ethylene production increases continuously during ripening, which is in stark contrast to the typical patterns observed in strawberries and wine [10,151]. The ethylene production of raspberry fruits is negatively correlated with hardness loss, and containers have been identified as the main source of ethylene [152,153]. In addition, experiments conducted on raspberry fruits in the white stage showed that the loss of fruit hardness was delayed during 1-MCP in vitro treatment when stored at 10 °C. These findings suggest that ethylene may play a partial role in regulating the softening process during raspberry ripening. In strawberries, the segregation of four *FaACS* genes and three *FaACO* genes has been recorded, with each gene exhibiting different expression patterns throughout the entire maturation process [154]. Similarly, various grape varieties such as Cabernet Sauvignon, Hamburg Muscat, and Thompson Seedless have shown that the presence of the *VvACO* gene is associated with an increase in ethylene, which occurs prior to its presence [23]. It is worth noting that during the development of strawberry fruit, the expression dynamics of two *FaACO* genes and the ethylene response sensor (*FaErs1*) have been observed, indicating a correlation between the expression of these genes and ethylene production.

#### 3.6.2. Ethylene Signal Transduction and Its Genetic Regulation

Many studies have emphasized changes in gene expression related to the ethylene signaling pathway during the ripening process of climacteric and non-climacteric fruits [155,156]. These genes include genes encoding ethylene receptor (ETR), ethylene response sensor (ERS), ethylene insensitive protein (*EIN*), and constitutive triple reactive protein (*CTR1*). *CTR1* is located on the endoplasmic reticulum (*ER*) membrane and serves as an intermediary between *ETRs* and *EIN2s*, playing a critical role in ethylene signaling transduction [155]. The *ETR* family, composed of transmembrane proteins in the ER, forms stable dimers with two disulfide bonds at the N-terminus upon ethylene binding. These receptors act as negative regulators of the ethylene pathway, blocking downstream signaling when ethylene is absent [155,156]. In transgenic tomatoes, early ripening was triggered by downregulating the SlETR4 gene [157].

In grape development, *VvETR2* expression increased at the start of ripening, while *VvETR1* remained consistently expressed. Similarly, *VvERS1* and *VvEIN4* exhibited higher expression levels shortly after anthesis. In strawberries, the expression of ethylene receptor genes FaEtr1, *FaErs1*, and *FaEtr2* coincided with increased ethylene production, with *FaEtr2* being predominantly expressed in ripe fruit. This suggests that even the low levels of ethylene produced during strawberry ripening are sufficient to activate ripening processes [158].

Further analysis revealed that the ethylene biosynthesis gene *FaSAMS1* and the signaling gene *FaCTR1* were transcriptionally induced in parallel with ethylene production during the fruit’s color change. The downregulation of these genes through the tobacco rattle virus-induced gene silencing (VIGS) system affects the production of red color, hardness, and ethylene. In addition, the application of ethephon (a synthetic ethylene-releasing agent) promotes the natural softening and color development of strawberries, partially restoring the biosynthesis of anthocyanins, although the effect on hardness is relatively small. This suggests that *FaCTR1* may play a role in regulating strawberry ripening, although it is unclear whether ethylene is involved in the early ripening stage of this non-climacteric fruit [159].

### 3.7. Jasmonates

Jasmonic acid (JA) and its bioactive isoleucine conjugate (JA Ile) are key signals in various plant stress responses, affecting root growth, seed germination, stamen development, and senescence [160].

#### 3.7.1. The Endogenous Effect of JA

A recent study suggests that endogenous JA levels (including JA Ile) and the expression of their biosynthetic genes decrease synchronously from the flowering to maturity stage of strawberry fruits [161]. During the early development of grape berries, elevated levels of JA and JA Ile were detected, followed by a sharp decline as the fruit matured [162]. This suggests that JA Ile may be related to the early fruit development of strawberries and grape berries.

It is worth noting that the accumulation pattern of anthocyanins (PA) during the development of strawberries and grape berries reflects the accumulation pattern of JA Ile, reaching its peak in the early stages of fruit development and decreasing with fruit ripening [163]. Observations have shown that the application of chemical inhibitors targeting JAR1 increases PA content. JAR1 is a key enzyme in JA Ile biosynthesis, indicating a potential link between the JA pathway and PA biosynthesis in strawberry fruit [164].

In Fragaria chiloensis fruit, exogenous methyl jasmonate (MeJA) significantly altered the expression of maturation-related genes, including genes involved in the biosynthesis of ethylene and jasmonic acid (JA) [165]. MeJA treatment also promoted anthocyanin accumulation by upregulating key genes in the anthocyanin biosynthesis pathway, such as chalcone synthase (*FcCHS*), chalcone flavonoid isomerase (*FcMHI*), flavanone 3-hydroxylase (*FcF3H*), dihydroflavonol 4-reductase (*FcDFR*), anthocyanin synthase (*Fc ANS*), and anthocyanin 3-O-glucosyltransferase (*Fc0FGT*). The increase in anthocyanin levels is also related to the upregulation of JA biosynthesis genes, including 13 lipoxygenase (*FcLOX*), propadiene oxide synthase (*FCAO*), and 12 oxo plant diesterase 3 (*FcOPR3*) [165]. Similarly, the application of *MeJA* in *Fragaria x ananassa* resulted in a significant increase in anthocyanin content and elevated levels of JA, JA Ile, and *MeJA.* JA has been shown to increase anthocyanin production in grape cell suspension, and the application of MeJA has increased the content of proanthocyanidins (PA) in two wine grape varieties [166]. In addition, research on raspberries emphasizes the role of jasmonate in enhancing phenylalanine ammonia lyase (PAL) activity, resulting in higher levels of polyphenolic compounds such as tannic acid, quercetin, and myricetin [167].

#### 3.7.2. Jasmonic Acid and Isoleucine Signaling Pathways

The biological processes regulated by JA Ile involve the activation of the jasmonic acid (JA) signaling pathway, in which the F-box protein *CORONATINE INSENSITIVE1* (*COI1*) forms a co-receptor with the jasmonic acid *ZIM-DOMAIN* (JAZ) protein. In the absence of sufficient JA Ile, JAZ repressors bind to transcription factors such as MYC2, inhibiting the expression of early JA responsive genes. When JA Ile levels increase, COI1 interacts with JAZ protein, leading to its ubiquitination and subsequent degradation by the 26S proteasome. This degradation releases MYC2 and other transcription factors, thereby activating JA responsive genes. Eleven JAZ members have been identified in grapevines, which respond to various stresses, hormones, and abiotic treatments [168]. Recent studies have shown that 12 potential JAZ proteins and two MYC transcription factor genes in strawberries are highly expressed during the flower and early fruit stages, corresponding to the downregulation of JA Ile observed during fruit development [161].

### 3.8. Brassinosteroids

Brassinosteroids (BR) are essential steroid plant hormones that regulate various plant processes, including cell division, elongation, vascular differentiation, flowering, pollen development, and photomorphogenesis [169]. They also play an important role in the fruit development and maturation of crops such as tomatoes, cucumbers, grapes, and strawberries [65,170]. Notably, BR has been shown to enhance anthocyanin biosynthesis, which contributes to the color development in non-climacteric fruits like strawberries and grapes. Additionally, BR influences ripeness by modulating key transcription factors that regulate fruit pigmentation and skin quality in these species [171].

#### 3.8.1. The Impact of BR on Maturity

In grape berries, the use of brassinolide (BR), especially brassinolide, has been found to enhance berry color and accelerate ripening. On the contrary, the use of BR biosynthesis inhibitor brasinazole (BZ) has the opposite effect. The enzyme BR 6-oxidase is responsible for converting 6-deoxytestosterone into active BR testosterone [172]. Overexpression of the grape BR 6-oxidase gene (*VvBR6OX1*) has been shown to restore the normal height of dwarf tomato plants lacking functional dwarf genes, enabling them to reach the same height as wild-type plants [172]. In strawberries, BL application has been observed to promote maturation and increase the expression of *FaBRI1* receptors. In addition, temporary inhibition of the *FaBRI1* gene leads to delayed maturation, resulting in the fruit clusters remaining white [170,173]. These findings highlight the critical role of BR signaling in the ripening of non-climacteric fruits.

#### 3.8.2. The Regulatory Role of BR and ABA

Brassinolide (BR) is a crucial plant hormone that regulates various growth processes, such as cell elongation and differentiation, by modulating gene expression related to cell wall loosening. Its ability to enhance cell division and expansion is vital for fruit size and shape development. BR is considered an initial signal of grape fruit ripening and may affect ethylene levels [129]. In terms of BR response genes, the late embryogenesis enriched (LEA) domain protein 1 (LDP1) gene is expressed in the early developmental stages of F. chilonensis and *F. vesca*, particularly in containers [174]. The promoter region of the LDP1 gene contains several motifs that respond to both BR and abscisic acid (ABA). Research has shown that the transient expression of *FcLDP* promoter GFP fusion is regulated by BR and ABA, highlighting the regulatory effects of these two hormones on FcLDP expression during fruit development in *F. chilonensis* [174].

### 3.9. Cytokinins

The study of cytokinins (CKs) in the development and maturation of non-climacteric fruits is an area that requires further exploration. Bombarely et al. (2016) identified two genes related to the CK signaling pathway in various *F. x ananassa* varieties from a fruit cDNA library, particularly the histidine phosphotransferase protein (*AHP*) and nuclear reaction regulatory factor (*ARR*) genes [175]. Afterwards, Kang et al. (2013) examined the transcriptome of *F. vesca* during the pre-fertilization and post-fertilization stages of fruit development and identified 17 differentially expressed genes (DEGs) associated with CK biosynthesis, signal transduction, and various fruit tissue degradation. The report indicates that CKs play a crucial role in the early stages of strawberry fruit development [176], just as they do in the early development of climacteric fruits such as tomatoes [177]. In grapes, the synthetic cytokinin forchlorfenuron (N-(2-chloro-4-pyridyl)-N’-phenylurea), known as CPPU, has been associated with increased berry weight, although it also led to reductions in sugar and anthocyanin content [178,179]. Notably, the only documented role of CKs in the Rubus genus suggests potential synergies between gibberellins (GAs) and CKs during flower induction in raspberries [180].

## 4. Conclusions

In conclusion, the molecular investigation of non-climacteric fruit models, such as grape, strawberry, and lesser-studied species like raspberry, has revealed that various phytohormones (including ABA, auxin, ethylene, and others) interact or regulate one another to influence multiple molecular and biochemical processes that determine fruit quality during the onset of ripening. Common genes such as *FW2.2*, *OVATE*, *SUN*, and *CLV-WUS* are frequently identified as crucial regulators of fruit shape across various fruit plants. These genes are involved in key processes like cell division and expansion, impacting overall fruit morphology. The growing body of research identifying and characterizing key genes associated with the signaling and perception of these hormones in grapes and strawberries could enhance our understanding of the ripening processes in other non-climacteric fruits, including under-researched species like Rubus. This knowledge may also guide strategies to improve post-harvest quality and food security.

## Data Availability

The authors declare no data to share.
